# Genetic susceptibility to PM_2.5_ exposure and transcriptional responses in pediatric asthma: insights from single-cell transcriptomics

**DOI:** 10.3389/fimmu.2026.1644537

**Published:** 2026-02-03

**Authors:** Jelte Kelchtermans, Huiqi Qu, Cuiping Hou, Frank Mentch, Sharon A. McGrath-Morrow, Hakon Hakonarson

**Affiliations:** 1 Department of Pediatrics, Children’s Hospital of Philadelphia, Philadelphia, PA, United States; 2 Perelman School of Medicine, University of Pennsylvania, Philadelphia, PA, United States

**Keywords:** asthma, gene-environment interaction, PM_2.5_, single-cell transcriptomics, susceptibility

## Abstract

**Background:**

Exposure to fine particulate matter (PM_2.5_) increases asthma severity and reduces glucocorticoid responsiveness in children, yet the molecular mechanisms underlying PM_2.5_ sensitivity remain unclear. We previously identified a PM_2.5_-sensitive asthma phenotype and developed a PM_2.5_ sensitivity polygenic risk score (sPRS) correlated with asthma exacerbations and lung function decline.

**Research question:**

We sought to determine whether genetic variants contributing to PM_2.5_ sensitivity converge on specific biological pathways or transcriptional regulators, and whether children with a high sPRS exhibit immune transcriptional signatures consistent with heightened PM_2.5_ susceptibility.

**Methods:**

Genes implicated by sPRS variants were mapped using regulatory annotation tools and evaluated for pathway and transcription factor target enrichment. Peripheral blood mononuclear cells (PBMCs) from high- and low-sPRS children matched on long-term ambient PM_2.5_ exposure were profiled using single-cell RNA sequencing. Donor-level pseudobulk differential expression was performed using a paired quasi-likelihood negative binomial framework, followed by exploratory pathway enrichment and perturbagen signature analyses.

**Results:**

sPRS-implicated genes were enriched for transcriptional regulators linked to SMAD2/3- and MAPK-associated signaling, suggesting TGF-β1-related pathway involvement. No genes reached false-discovery-rate-adjusted significance at the donor level in this small, matched cohort. However, secondary pathway-level analyses demonstrated concordant enrichment across multiple immune populations in inflammatory and stress-response signaling programs previously linked to PM_2.5_ exposure. Perturbagen signature analyses likewise highlighted small-molecule regulators of TGF-β1-associated pathways.

**Interpretation:**

These integrative genomic and transcriptomic analyses nominate TGF-β1-SMAD/MAPK signaling as a biologically plausible axis of genetic susceptibility to PM_2.5_ in pediatric asthma. Given the modest sample size and indirect nature of enrichment-based inference, these findings should be considered hypothesis-generating and motivate targeted functional validation.

## 1 Introduction

Fine particulate matter (PM_2.5_) increases both the incidence and severity of pediatric asthma and is known to impair corticosteroid responsiveness (1–7). Despite this, PM_2.5_ exposure remains a global problem with 99% of the global population exposed to concentrations exceeding World Health Organization guideline levels (8). Given this widespread exposure, interventions that mitigate the adverse health effects of PM_2.5_ exposure in children with asthma are urgently needed. Unfortunately, developing interventions with strong reproducible effects on asthma outcomes has proven challenging, likely in part due to asthma heterogeneity and complex gene-environment interactions (9–12).

Mechanisms to stratify patients based on their susceptibility to PM_2.5_ exposure may help us overcome these issues. Incorporating sensitivity to PM_2.5_ in trial design may decrease statistical noise and support outcome replication as it avoids issues related to significant variability in individual responses to air pollution. To this end, we previously described a PM_2.5_-sensitive pediatric asthma phenotype and developed a PM_2.5_ sensitivity polygenic risk score (sPRS), which correlates with asthma exacerbations and lung function in exposed children (13, 14).

Beyond risk stratification, these findings raise the question if genetic variants included in this sPRS converge on specific biological pathways or cell types that may be implicated in PM_2.5_ susceptibility, and whether such convergence may provide mechanistic insight into differential responses to air pollution. Single-cell transcriptomics studies previously identified cell-specific responses to PM_2.5_ exposure, with inflammatory activation occurring in monocytes, T cells, and innate lymphoid cells (15). Other studies have demonstrated that exposure to PM_2.5_ can lead to significant gene expression changes in lung fibroblasts (16). Additionally, PM_2.5_ exposure has been shown to alter immune cell communication networks and to activate oxidative stress pathways (17). However, whether genetic variants associated with PM_2.5_ sensitivity influence the transcriptional perturbation of PM_2.5_ remains unknown.

To address this knowledge gap, we applied an integrative approach combining genetic mapping, single-cell transcriptomics, and functional enrichment analysis to decipher the molecular pathways associated with variants included in the sPRS. First, we mapped sPRS variants to genes using regulatory annotation tools, assessing whether these genes converge on specific biological pathways or are disproportionately influenced by key transcriptional regulators. Next, we performed single-cell RNA sequencing (scRNA-seq) on peripheral blood mononuclear cells (PBMCs) from patients stratified by sPRS to determine whether individuals with high sPRS exhibit distinct transcriptional responses to PM_2.5_ exposure at the cellular level. Finally, we leveraged pathway enrichment and in silico perturbagen signature analyses to nominate candidate regulatory networks for future functional investigation.

## 2 Methods

### 2.1 PM_2.5_ sensitivity polygenic risk score

As previously reported, our genotype data were generated on genotyping arrays from Illumina, using standard quality assurance cut-offs (detailed in the supplementary methods description) (14). Ancestry was assigned based on principal component analysis. Our previous publication outlines how we defined a PM_2.5_ sensitive pediatric asthma phenotype, assessed which SNPs are associated with this PM_2.5_ sensitivity in children with asthma, and used this data to create a sPRS (14).

For the current study, we use the Open Targets Genetics Variant to Gene (V2G) tool to assess which genes are implicated by the variants included in the sPRS (18–20). We then use the Metascape and Enrichr tools to assess if any biological pathways are significantly enriched for members of this gene set (21–25). Q-values were adjusted via the Benjamini-Hochberg procedure to account for multiple testing (26, 27). Enriched terms were hierarchically clustered based on Kappa scores, with clusters defined by a similarity threshold of > 0.3 (28). Transcription factor target enrichment was further assessed using the ChEA 2022 dataset via Enrichr, and Metascape was used to perform complete pathway analysis for the target genes of enriched transcription factors.

### 2.2 Single cell RNA sequencing

Patients with asthma and at least one year of follow-up data after their initial asthma diagnosis were selected from the biobank at [*Institution censored for review*] as described previously (14). All subjects have provided informed consent to both genomic analysis and EMR mining as approved by our institutional IRB. Asthma cases were identified using a previously validated electronic medical record (EMR)-derived phenotype (29). To avoid confounding our results by population structure, only patients from our largest ancestry group (AFR) were selected for this study. Within this group, eight patients with an ancestry-specific sPRS z-score above one were matched with eight patients with an ancestry-specific sPRS z-score below negative one. Patients were matched by birth year, sex, sample collection month, and by living in a non-high-income (median household income as determined using census data <75^th^ percentile) area with frequent air pollution exposure (incidence of air quality index >50 >75^th^ percentile). This matching strategy was designed to ensure similar long-term air pollution exposure while minimizing confounding by holding key environmental and demographic variables constant.

Please refer to the supplementary methods section for details regarding sample processing, sequencing, and data processing approach.

Uniform Manifold Approximation and Projection (UMAP) was used to define and visualize cell clusters (30). For cell annotation, SingleR was applied using the celldex::DatabaseImmuneCellExpression reference (31). To assess the robustness of cell-type assignments, complementary validation analyses were performed using two independent immune reference atlases (MonacoImmuneData and Blueprint/ENCODE) (32, 33).

For differential expression analyses, gene-level counts were aggregated at the donor level within each of the 15 SingleR-defined cell types to generate pseudobulk expression matrices. Donor-level differential expression was tested using edgeR’s negative binomial quasi-likelihood framework, with matched pair included as a blocking factor to account for the paired study design (34). Genes were considered differentially expressed if they passed a Benjamini-Hochberg false discovery rate (FDR) threshold of 0.05.

Given the modest sample size and corresponding limitations in power at the donor level, we additionally conducted secondary, exploratory analyses to provide biological context for the observed expression patterns. Within each annotated cell type, genes meeting a nominal significance threshold of P < 0.05 in the pseudobulk analysis were assembled into cell-type–specific gene sets for downstream enrichment analyses. These gene sets were evaluated for enrichment of previously reported PM_2.5_-associated transcriptional signatures using the Rummagene tool on the Enrichr platform (query term “PM_2.5_”), with enrichment significance evaluated using adjusted P values. Pathway and biological process enrichment analyses were performed using Metascape, with cell type-specific gene lists submitted together, allowing enrichment to be computed per list and recurrent pathway themes to be identified across immune compartments.

We also performed perturbagen enrichment analyses using the LINCS L1000 Signature Search (L2S2) platform accessed through Enrichr. For each immune cell type, nominally significant genes were partitioned into up-regulated and down-regulated sets and submitted separately to identify perturbagens whose transcriptional signatures were either concordant (“Up”) or inversely aligned (“Down”) with the observed expression patterns. All secondary analyses were prespecified as exploratory and hypothesis-generating.

### 2.3 Signal localization

To explore the potential pulmonary relevance of sPRS-associated variants, we queried the GTEx database to identify expression quantitative trait loci (eQTL) in lung tissue. (See acknowledgement section) We then used the LungMAP portal to identify pulmonary cell types known to express genes affected by these pulmonary eQTLs (35, 36). Similarly, as analyses results implicated transforming growth factor beta (TGF-β1), we used LungMAP to identify pulmonary cell types known to express TGF-β1.

## 3 Results

### 3.1 PM_2.5_ sensitivity polygenic risk score

**Figure 1:**
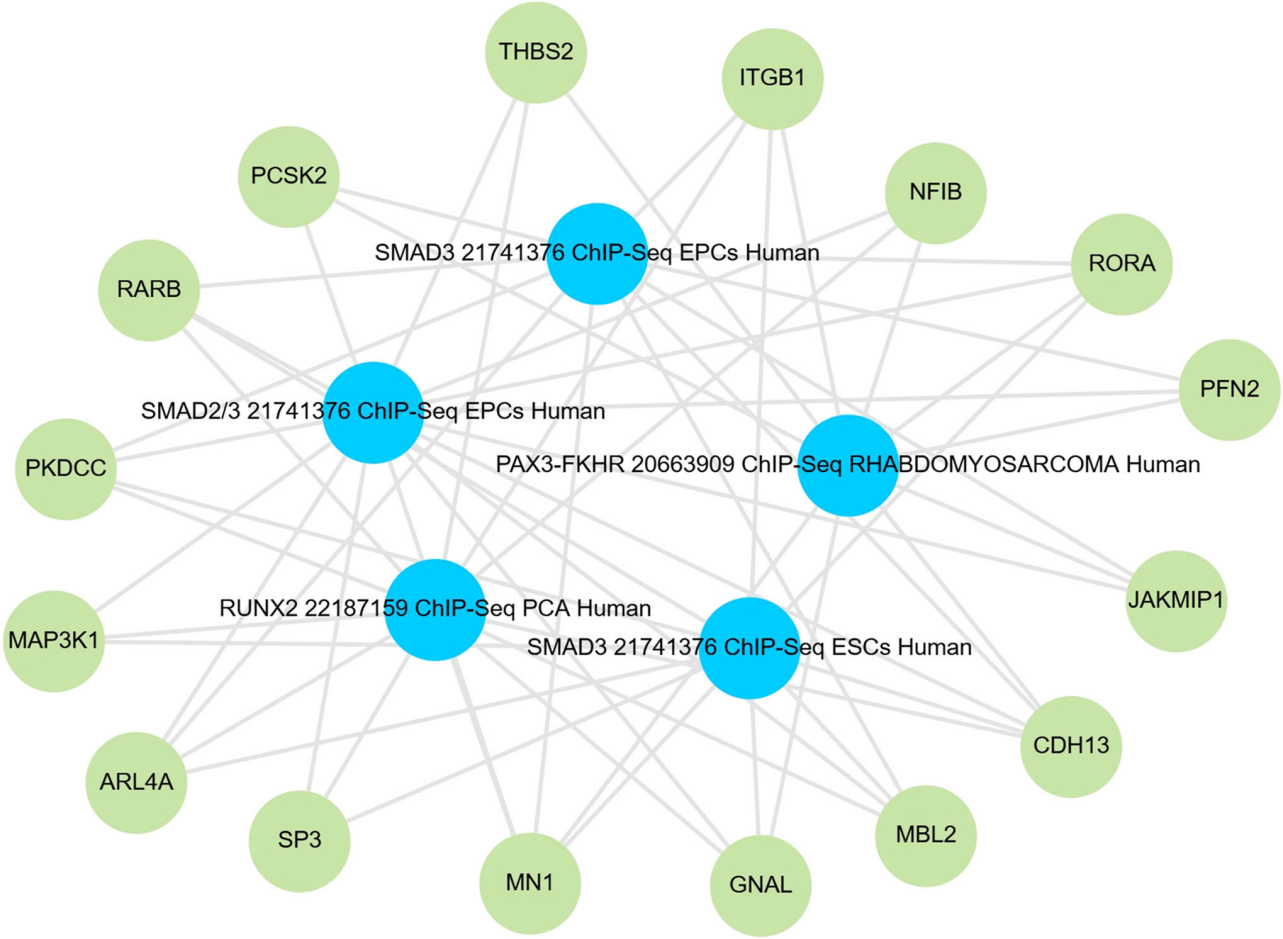
Network representation of enriched transcription factor gene sets and associated sPRS-linked genes. Variant-to-gene mapping using the V2G tool successfully linked 43 of the 44 resolvable sPRS variants to corresponding genes (**Table 1**). Among these, gene sets of five transcription factors, derived from the ChEA 2022 dataset, were significantly enriched: RUNX2 in prostate cancer cells (q-value = 3.19 × 10^-6^), SMAD2/3 in embryonic progenitor cells (q-value =2.18 × 10^-5^), PAX3-FKHR in rhabdomyosarcoma cells (q-value = 7.5 × 10^-5^), SMAD3 in embryonic progenitor cells (q-value = 2.67 × 10^-4^), and SMAD3 in embryonic stem cells (q-value = 3.34 × 10^-4^). In the network, blue nodes represent these transcription factors, while green nodes represent the sPRS-linked genes that are targets of these factors, with edge connections indicating regulatory interactions.

Of the 52 variants comprising the sPRS, 44 were resolvable to rsIDs, and 43 of these were successfully linked to candidate genes using the V2G tool (**Table 1**, **Supplementary Table 1**). Notably, a disproportionate number of these genes were identified as targets of the transcription factors RUNX2, SMAD2/3, or PAX3-FKHR (q-value 3.19 x 10^-6^, 2.18 x 10^-5^, and 7.5 x 10^-5^, respectively, **Figure 1**) (37, 38). Although the target networks for RUNX2 and PAX3-FKHR were originally defined in cancer contexts and thus may not be directly relevant to pediatric asthma, these associations may suggest the involvement of similar downstream cascades.

**Table 1:** Variant-to-gene mapping results for sPRS variants.

Variant	Gene symbol	Description
rs1887910	AMY1C	amylase alpha 1C
rs7529723	NEK7	NIMA related kinase 7
rs149463320	EDARADD	EDAR associated via death domain
rs6548226	ALKAL2	ALK and LTK ligand 2
rs373472462	None identified	
rs74534741	FEZ2	fasciculation and elongation protein zeta 2
rs4245776	PKDCC	protein kinase domain containing, cytoplasmic
rs35384502	LIMS1	LIM zinc finger domain containing 1
rs76504660	SP3	Sp3 transcription factor
rs7616736	RARB	retinoic acid receptor beta
rs13070250	TAFA4	TAFA chemokine like family member 4
rs9836522	PFN2	profilin 2
rs34333139	JAKMIP1	janus kinase and microtubule interacting protein 1
rs201561293	USP53	ubiquitin specific peptidase 53
rs289012	MAP3K1	mitogen-activated protein kinase kinase kinase 1
rs7736051	COX7C	cytochrome c oxidase subunit 7C
rs11965772	DCDC2	doublecortin domain containing 2
rs61448283	THBS2	thrombospondin 2
rs2033605	ARL4A	ARF like GTPase 4A
rs13221603	AGMO	alkylglycerol monooxygenase
rs6969926	CALN1	calneuron 1
rs148380793	WNT16	Wnt family member 16
rs10155910	TMEM140	transmembrane protein 140
rs7838541	NKX6-3	NK6 homeobox 3
rs112795425	YTHDF3	YTH N6-methyladenosine RNA binding protein F3
rs297554	TP53INP1	tumor protein p53 inducible nuclear protein 1
rs10961558	NFIB	nuclear factor I B
rs7853136	ROR2	receptor tyrosine kinase like orphan receptor 2
rs796841295	ITGB1	integrin subunit beta 1
rs12218358	MBL2	mannose binding lectin 2
rs12708352	PPFIBP2	PPFIA binding protein 2
rs2199664	LGR4	leucine rich repeat containing G protein-coupled receptor 4
rs6590627	NTM	neurotrimin
rs10771491	FAR2	fatty acyl-CoA reductase 2
rs74487335	DOCK9	dedicator of cytokinesis 9
rs2099587	RORA	RAR related orphan receptor A
rs763727	CDH13	cadherin 13
rs8078468	GOSR2	golgi SNAP receptor complex member 2
rs116380170	GRIN2C	glutamate ionotropic receptor NMDA type subunit 2C
rs79507261	GNAL	G protein subunit alpha L
rs4802432	PLA2G4C	phospholipase A2 group IVC
rs6111430	PCSK2	proprotein convertase subtilisin/kexin type 2
rs6517487	ETS2	ETS proto-oncogene 2, transcription factor
rs7290038	MN1	MN1 proto-oncogene, transcriptional regulator

Variant-to-gene mapping was conducted using the V2G tool, which linked 43 of the 44 resolvable variants in the sPRS to corresponding genes. The table details the variant identifiers and their assigned gene targets.

To further investigate this, we conducted a Metascape enrichment analysis on the gene sets corresponding to RUNX2 and PAX3-FKHR target genes. This analysis revealed significant enrichment of the term “GO:0043408 regulation of MAPK cascade” for the target genes of both RUNX2 and PAX3-FKHR (P = 4.56 x 10–^17^ and P = 2.72 x 10^-11^, respectively, **Supplementary Table 2**). Taken together, the genes implicated by the sPRS converge around the SMAD and MAPK pathways.

### 3.2 Gene expression analysis

Cell-type annotations were validated across independent reference datasets after harmonization to major peripheral blood immune lineages, demonstrating consistent classification of the major immune populations included in downstream analyses. Donor-level pseudobulk differential expression analysis did not identify statistically significant gene-level differences that passed false discovery rate correction in this small, matched sample. To provide biological context for cell-level transcriptional variation, we therefore examined transcriptional patterns across immune populations in a prespecified secondary analysis.

**Figure 2:**
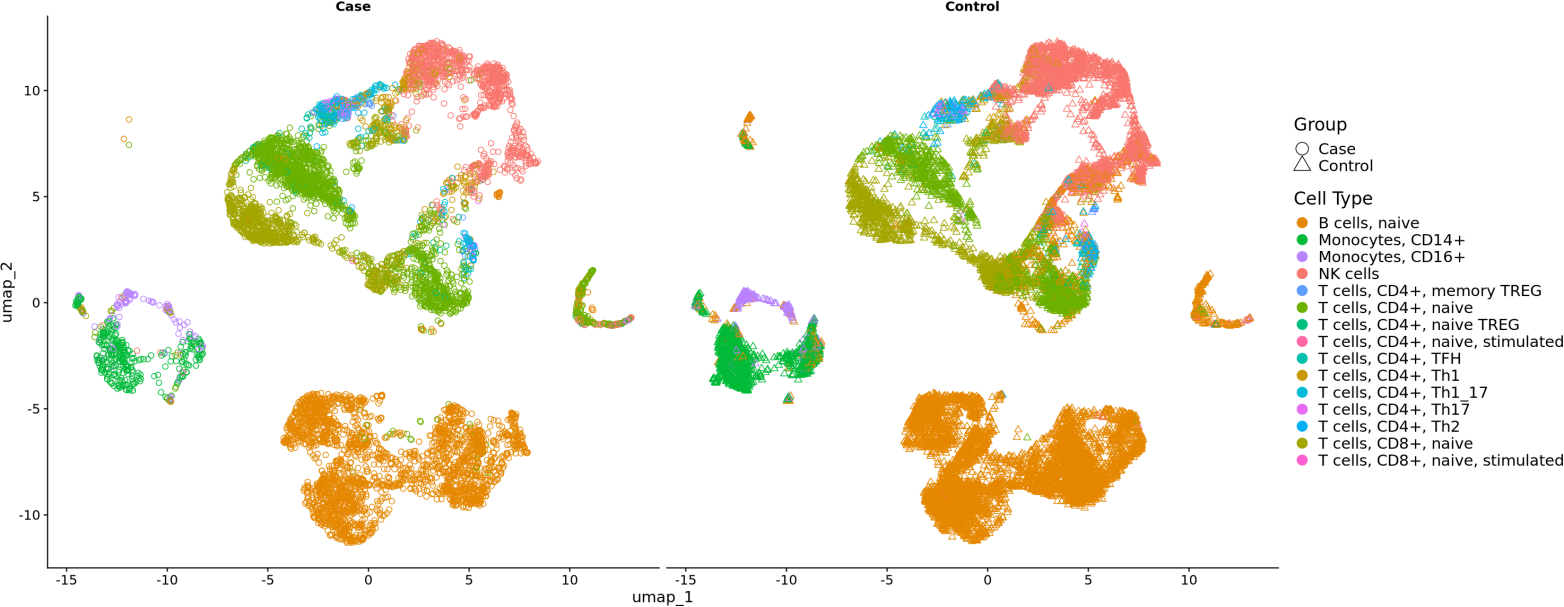
UMAP visualization of single-cell RNA sequencing data from the first matched pair of case and control subjects. Immune cell populations are displayed, with cases (circles, left) and controls (triangles, right) split by group. Each point represents a single cell, colored by cell type.

In this secondary analysis, 14 out of 15 cell types had genes meeting the nominal significance threshold (**Figure 2**, **Supplementary Table 3**). These gene sets showed overlap with gene sets previously found to have differential expression after PM_2.5_ exposure (**Table 2**, **Supplementary Table 4**). Because cases and controls were matched on ambient PM_2.5_ exposure but selected based on sPRS, these findings are consistent with the hypothesis that individuals with a high sPRS may exhibit greater transcriptional perturbation for a given exposure.

**Table 2:** Overlap of differentially expressed genes with *in vitro* PM_2.5_-responsive transcripts.

Cell type	Paper	Adjusted p-value
Naïve B Cells	PMC3275518	0.03
	PMC6906712	0.04
CD14 Positive Monocytes	PMC3275518	0.00712
	PMC3275518	0.0392
	PMC3275518	0.042
NK-cells	PMC3275518	1.86E-09
	PMC3275518	6.65E-09
	PMC3275518	2.80E-07
	PMC3275518	0.000047
	PMC3275518	0.00298
	PMC3275518	0.0044
	PMC8710082	0.00512
CD4 Positive T follicular helper cells	PMC3275518	0.0237
CD4 positive T helper cell population with combined Th1 and Th17 features	PMC3275518	0.00198
	PMC3275518	0.00365
	PMC3275518	0.00472
	PMC8823376	0.00925
	PMC3275518	0.0158
CD4 positive Th1 cells	PMC3275518	0.0000186
	PMC3275518	0.0000635
	PMC3275518	0.00365
	PMC3275518	0.0244
	PMC12588579	0.0413
CD8 positive naïve T-cells	PMC3275518	0.000985
	PMC8823376	0.0126
	PMC12588579	0.0277

This table summarizes the significance of the overlap between genes differentially expressed in our immune cell subsets and those previously identified as transcriptionally responsive to *in vitro* PM_2.5_ exposure. For details regarding the specific genes that overlap please refer to **Supplementary Table 4**. The observed overlap is consistent with enhanced transcriptional perturbation in high-sPRS individuals despite matching on ambient PM_2.5_ exposure.

To characterize recurrent biological themes across immune populations, we next evaluated pathway and process enrichment using Metascape, with multiple-testing correction applied. Enriched terms clustered into three broad functional superfamilies (**Supplementary Table 5**, **Table 3**).

**Table 3:** Most significant hierarchically clustered biological processes and pathways enriched for differentially expressed genes.

Superfamily	Term	Description	Log(q-value)
Transcription and protein synthesis	GO:0006886	intracellular protein transport	-33.27
	GO:0031399	regulation of protein modification process	-31.92
	R-HSA-2408557	Selenocysteine synthesis	-24.15
	R-HSA-9675108	Nervous system development	-25.66
	R-HSA-5653656	Vesicle-mediated transport	-25.47
	GO:0051248	negative regulation of protein metabolic process	-24.44
Immune activation	GO:0045321	leukocyte activation	-49.43
	R-HSA-2262752	Cellular responses to stress	-46.79
	GO:0050778	positive regulation of immune response	-38.61
	GO:0001819	positive regulation of cytokine production	-38.03
	GO:0002694	regulation of leukocyte activation	-37.23
	GO:0080135	regulation of cellular response to stress	-35.71
	R-HSA-1280218	Adaptive Immune System	-32.72
	R-HSA-9679506	SARS-CoV Infections	-26.93
	GO:0071345	cellular response to cytokine stimulus	-26.26
Signaling pathways	R-HSA-9006934	Signaling by Receptor Tyrosine Kinases	-31.37
	GO:0007169	cell surface receptor protein tyrosine kinase signaling pathway	-23.95
	GO:0043408	regulation of MAPK cascade	-24.58
	R-HSA-5683057	MAPK family signaling cascades	-13.58
	R-HSA-6785807	Interleukin-4 and Interleukin-13 signaling	-8.98

Enrichment analysis was conducted using the Metascape tool with default parameters (p-value < 0.01, minimum count of 3, enrichment factor > 1.5). P-values were computed via the cumulative hypergeometric distribution and adjusted to q-values using the Benjamini-Hochberg procedure. Enriched terms were hierarchically clustered based on Kappa scores (similarity threshold > 0.3), yielding twenty clusters that were further grouped into three overarching superfamilies: Transcription and Protein Synthesis, Immune Activation, and Signaling Pathways. This table displays representative terms from each superfamily, along with their descriptions and log-transformed q-values, reflecting the strength of the enrichment associations, please refer to **Supplementary Table 5** for full results.

The first superfamily encompassed pathways related to transcription and protein synthesis, including “intracellular protein transport” (P = 3.55 x 10-15), “regulation of protein modification process” (P = 1.37 x 10-14), “Selenocysteine synthesis” (P = 3.26 x 10-11), “Nervous system development” (P = 7.17 x 10-12), “Vesicle-mediated transport” (P = 8.71 x 10-12), “negative regulation of protein metabolic process” (P = 2.42 x 10-11).

The second superfamily centered on immune system activation, with significant enrichment observed for terms such as “leukocyte activation” (P = 3.42 x 10-22), “Cellular responses to stress” (P = 4.79 x 10-21), “positive regulation of immune response” (P = 1.71 x 10-17), “positive regulation of cytokine production” (P = 3.04 x 10-17), “regulation of leukocyte activation” (P = 6.75 x 10-17), “regulation of cellular response to stress” (P = 3.11 x 10-16), “Adaptive Immune System” (P = 6.18 x 10-15), “SARS-CoV Infections” (P = 2.01 x 10-12).

Finally, the third superfamily was comprised of various signaling pathways. Specifically, “Interleukin-4 and Interleukin-13 signaling” (P = 0.0001), “MAPK family signaling cascades” (P = 1.27 x 10-6), “Signaling by Receptor Tyrosine Kinases” (P = 2.38 x 10-14), “cell surface receptor protein tyrosine kinase signaling pathway” (P = 3.99 x 10-11), “cellular response to cytokine stimulus” (P = 3.93 x 10-12), “regulation of MAPK cascade” (P = 2.11 x 10-11).

To further contextualize these immune transcriptional signatures, we queried the LINCS L1000 Signature Search (L2S2) database to identify small-molecule perturbagens whose induced transcriptional profiles were either concordant (“UP-UP” or “DOWN-DOWN”) or inversely aligned (“UP-DOWN” or “DOWN-UP”) with the observed expression patterns across immune cell types. Perturbagens were prioritized based on replication of enrichment across multiple immune populations, with statistical strength used as a secondary criterion.

Several compounds demonstrated recurrent inverse alignment with the observed gene expression signatures (UP-DOWN quadrant), most notably tretinoin, BMS-387032 (a CDK inhibitor), and mitoxantrone. Each of these agents showed significant enrichment across 7 of the 10 immune cell types in which an L2S2 hit was observed in the UP–DOWN quadrant, with minimum adjusted P values ranging from 4.4 × 10–^6^ to 4.4 × 10^-16^. Compounds demonstrating concordant alignment (UP-UP quadrant) across the largest number of immune populations included BRD-K08177763, niclosamide, and tozasertib, each enriched in 7 of 10 cell types with a significant UP-UP signature (minimum adjusted P values between 4.5 × 10–^9^ and 5.3 × 10^-5^). In contrast, relatively few perturbagens showed consistent alignment in the DOWN-UP or DOWN-DOWN quadrants, and these signals were limited to two contributing cell types. Consistent with the exploratory nature of this analysis, these results are interpreted as hypothesis-generating pathway concordance signals rather than evidence of therapeutic relevance.

### 3.3 Signal localization

We next examined the impact of the sPRS variants by assessing which of them are expression quantitative trait loci (eQTL) in lung tissue. We found that rs9836522 is an eQTL for *PFN2* with the allele conferring higher PM_2.5_ sensitivity (the ‘risk allele’) associated with higher PFN2 expression (P = 0.01, **Supplementary Figure 1**). Furthermore, the risk alleles for rs13221603 and rs10155910 were associated with lower expression of *AGMO* and *TMEM140* respectively (P = 0.021 and P = 0.023 respectively) in lung tissue. To pinpoint the cellular context of these associations, we queried the Human Lung Reference Cell Atlas (version 1.0). *PFN2* expression was predominantly observed in ionocytes, pulmonary neuroendocrine cells (PNEC), and Deuterosomal cells (P = 3.18 x 10^-76^, P = 1.20 x 10^-65^, and P = 3.09 x 10–^57^ respectively). Similarly, *AGMO* expression was enriched in PNEC and systemic venous endothelial cells (SVEC) (P = 2.29 x 10–^5^ and P = 0.0001), while *TMEM140* expression was most prominent in lymphatic endothelial cells and CAP2 cells (P = 3.80 x 10–^29^ and 3.63 x 10^-18^).

Since signaling pathways implicated by the sPRS (SMAD2/3 and MAPK) and by the gene expression analysis (, MAPK, Receptor Tyrosine Kinase receptors, and IL4/IL13) converge around TGF-β1, we also assessed its expression in lung tissue. This analysis revealed that TGF-β1 is preferentially expressed by interstitial macrophages, patrolling monocytes, inflammatory monocytes, cDC1 cells, NK cells, and pDC cells (P = 3.98 x 10^-25^, P = 7.94 x 10^-24^, P = 7.08 x 10^-12^, P = 2.09 x 10^-11^, P = 3.39 x 10^-11^, and P = 4.27 x 10^-10^, respectively). Additionally, we assessed if differentially expressed genes overlapped with gene-sets previously linked to TGF-β1. This was found to be the case for Naïve B-Cells (adjusted P = 5.14 x 10^-20^), CD14^+^ monocytes (adjusted P = 7.61 x 10^-9^), CD16^+^ monocytes (adjusted P = 4.46 x 10^-8^), CD4^+^ naïve T cells (adjusted P = 4.75 x 10^-112^), CD8^+^ naïve T cells (adjusted P = 2.80 x 10^-18^), and NK cells (adjusted P = 2.19 x 10^-37^) (39, 40).

## 4 Discussion

In this work, we combined a PM_2.5_ sPRS with single‐cell transcriptomics to explore how genetic predisposition influences cellular responses to PM_2.5_ in pediatric asthma. By matching high‐ and low‐sPRS children on long term ambient PM_2.5_ exposure and key demographic factors, our design minimizes non‐genetic confounding. Under these conditions, exploratory analyses suggested broader and more pronounced transcriptional perturbation in multiple immune populations, despite equivalent long term pollution levels, consistent with genetic susceptibility being associated with amplified molecular perturbation upon PM_2.5_ exposure.

Mapping sPRS variants to genes via the Open Targets V2G tool linked 43 of the 44 resolvable variants to specific gene targets. These genes were enriched for targets of SMAD2/3 and RUNX2/PAX3-FKHR transcription factors. Although RUNX2 and PAX3-FKHR were originally characterized in oncogenic contexts, their shared enrichment for “regulation of MAPK cascade” implies that SMAD/MAPK crosstalk may play a role in PM_2.5_ sensitivity (37, 38). Prior rodent models have shown that SMAD3 mediates PM_2.5_-induced fetal lung injury, and that SMAD3 inhibition reduces fibrosis in asthma models (41, 42). Furthermore, the inclusion of a *PFN2* eQTL in the sPRS is notable since PFN2 can epigenetically regulate SMAD2/3 activity (43). In asthma and COPD, TGF-β1 interconnects SMAD and MAPK pathways to drive airway remodeling, and PM_2.5_ itself induces TGF-β1 expression in lung tissue (44–47). Together, these observations nominate TGF-β1-SMAD/MAPK signaling as a plausible pathway through which genetic variation may modulate PM_2.5_ sensitivity in asthma.

Donor-level pseudobulk analysis did not identify statistically significant gene-level differences after multiple-testing correction, underscoring the limited power of this small, matched cohort to support definitive gene-level inference. However, predefined secondary analyses identified consistent transcriptional patterns across several immune populations. In these secondary analyses gene sets showed concordant overlap with transcripts previously reported to respond to *in vitro* PM_2.5_ exposure. Pathway enrichment analyses clustered these gene sets into three broad functional superfamilies: transcription and protein synthesis, immune activation, and signaling cascades including MAPK, Tyrosine Kinase receptors, and IL-4/IL-13 pathways.

Notably, these signaling programs intersect extensively with TGF-β1 biology. TGF-β receptors are serine/threonine kinase receptors that canonically signal via SMAD2/3, but they also activate MAPK cascades and cooperate with receptor tyrosine kinase (RTK) pathways and IL-4/IL-13-driven cytokine signaling to regulate fibrosis, airway remodeling, and immune activation (48, 49). In this context, enrichment of MAPK, receptor tyrosine kinase, and IL-4/IL-13 pathways in our exploratory analyses is biologically consistent with a TGF-β1-centered model of genetic sensitivity to PM_2.5_, while not constituting direct evidence of pathway activation.

Similarly, the most consistent inverse signatures (UP-DOWN quadrant) include tretinoin and mitoxantrone, both of which have prior links to TGF-β-regulated remodeling pathways. In experimental glomerulonephritis, retinoids such as all-trans retinoic acid and isotretinoin attenuate TGF-β1 overexpression and limit glomerular collagen III/IV accumulation *in vivo*, consistent with an ability to dampen TGFβ1 driven profibrotic remodeling (50). Mitoxantrone has been shown to suppress TGF-β–induced type I collagen synthesis in primary human dermal fibroblasts by inhibiting SMAD3 phosphorylation (51). In parallel, niclosamide, which emerged among the top concordant UP-UP signatures, has been reported to attenuate TGF-β1-driven fibroblast activation and extracellular matrix accumulation across multiple fibrotic models via modulation of MAPK-ERK and related pathways. Taken together, these observations suggest that our in-silico screen preferentially identifies perturbagens that modulate TGF-β1-centric profibrotic and stress-response programs, although we interpret these findings as exploratory pathway concordance rather than evidence of therapeutic suitability for asthma.

Finally, our localization analysis identified preferential expression of TGF-β1 in immune cells, particularly interstitial macrophages, patrolling monocytes, inflammatory monocytes, cDC1 cells, NK cells, and pDC cells, potentially supporting the hypothesis that these cells mediate the initial pulmonary response to PM_2.5_ exposure. As key sentinels of the immune system, these cells are well-positioned to detect and react to inhaled pollutants. In contrast, the genes implicated by eQTLs in the sPRS (*PFN2*, *AGMO*, and *TMEM140*) were primarily expressed in structural and epithelial cell types, including ionocytes, PNECs, Deuterosomal cells, SVECs, lymphatic endothelial cells, and CAP2 cells. This may indicate a two-step mechanism, in which PM_2.5_ exposure first activates a TGF-β1 immune response, with genetic susceptibility to PM_2.5_ sensitivity concentrated in the epithelial and endothelial response to this stimulus.

Our study has several limitations. First, while our cohort includes a well-characterized pediatric asthma population, the relatively small sample size in the scRNA-seq experiment limits our ability to detect subtle transcriptional changes in rarer immune cell populations. Second, air-pollution exposure was assigned at the ZIP-code level. Community level exposure is widely used in epidemiologic research and has been consistently associated with asthma morbidity and other clinically relevant outcomes (52–62). However, as with any population-level exposure measure short of personal monitoring, some misclassification is expected due to differences in time spent outdoors, indoor microenvironments, and individual breathing patterns. This type of measurement error is likely to be predominantly non-differential with respect to immune-cell transcriptional state and would therefore bias associations toward the null. Third, while our integrative approach may suggest a role for TGF-β1 signaling in PM_2.5_ sensitivity, functional validation in cell or animal models is necessary to confirm this hypothesis. Also, our study focused on individuals of African ancestry, and future studies should examine whether similar genetic-environment interactions occur in other populations. In addition, the signal localization analyses using GTEx and LungMAP provide indirect evidence regarding potential lung relevance of the genetic associations and are intended to support biological plausibility rather than establish lung-specific mechanisms. Finally, the pathway-level interpretation of cell-type-specific transcriptional signatures relied on enrichment platforms such as Rummagene/Enrichr and Metascape. These approaches are inherently influenced by database curation, keyword-based retrieval, and overlapping gene-set structure, and are therefore best interpreted as exploratory. Consistent with this, we emphasize recurrent biological themes rather than individual gene-level findings, and we interpret these results as pathway-level concordance signals rather than confirmatory evidence of causal mechanisms.

In conclusion, this study integrates genetic mapping with single-cell transcriptomics to examine how genetic predisposition may modify immune transcriptional responses to PM_2.5_ in pediatric asthma. Although no donor-level cell-type differential expression reached false discovery rate significance, secondary, pathway-level analyses identified convergent enrichment of TGF-β1-SMAD/MAPK signaling program across immune cell populations. These findings provide exploratory, pathway-level context for prior epidemiologic observations linking polygenic sensitivity to PM_2.5_ with worse asthma outcomes and reduced glucocorticoid responsiveness and suggest biologically plausible axes for further investigation. Accounting for inter-individual genetic variation in these pathways may also help explain heterogeneity observed in prior therapeutic studies targeting TGF-β1 signaling (44).

Given the indirect nature of enrichment-based inference and the modest sample size, these results should be interpreted as hypothesis-generating rather than confirmatory. Expansion of this work to larger, multi-ancestry cohorts, incorporation of longitudinal exposure metrics, and integration of LD expansion, statistical fine-mapping, and eQTL colocalization will be important to refine putative effector genes and mechanisms. Ultimately, this line of inquiry may help enable precision-medicine strategies aimed at mitigating the health burden of air-pollution exposure among genetically susceptible children with asthma.

## Data Availability

The datasets presented in this study can be found in online repositories. The names of the repository/repositories and accession number(s) can be found below: GSE300837 (GEO).

## References

[1] Ni R Su H Burnett RT Guo Y Cheng Y . Long-term exposure to PM2.5 has significant adverse effects on childhood and adult asthma: A global meta-analysis and health impact assessment. One Earth. (2024) 7:1953–69. doi: 10.1016/j.oneear.2024.09.022

[2] Fan J Li S Fan C Bai Z Yang K . The impact of PM2.5 on asthma emergency department visits: a systematic review and meta-analysis. Environ Sci pollut Res. (2016) 23:843–50. doi: 10.1007/s11356-015-5321-x, PMID: 26347419

[3] Burbank AJ Sood AK Kesic MJ Peden DB Hernandez ML . Environmental determinants of allergy and asthma in early life. J Allergy Clin Immunol. (2017) 140:1–12. doi: 10.1016/j.jaci.2017.05.010, PMID: 28673399 PMC5675123

[4] Hehua Z Qing C Shanyan G Qijun W Yuhong Z . The impact of prenatal exposure to air pollution on childhood wheezing and asthma: A systematic review. Environ Res. (2017) 159:519–30. doi: 10.1016/j.envres.2017.08.038, PMID: 28888196

[5] Khreis H Kelly C Tate J Parslow R Lucas K Nieuwenhuijsen M . Exposure to traffic-related air pollution and risk of development of childhood asthma: A systematic review and meta-analysis. Environ Int. (2017) 100:1–31. doi: 10.1016/j.envint.2016.11.012, PMID: 27881237

[6] Asthma GIf . GINA MAIN REPORT. Fontana, WI. (2024).

[7] Lewis TC Robins TG Mentz GB Zhang X Mukherjee B Lin X . Air pollution and respiratory symptoms among children with asthma: vulnerability by corticosteroid use and residence area. Sci Total Environ. (2013) 448:48–55. doi: 10.1016/j.scitotenv.2012.11.070, PMID: 23273373 PMC4327853

[8] Institute HE . State of global air 2024. Special report. Boston, MA. (2024).

[9] Beasley R Semprini A Mitchell EA . Risk factors for asthma: is prevention possible? Lancet (London England). (2015) 386:1075–85. 10.1016/S0140-6736(15)00156-7 26382999

[10] Colilla S Nicolae D Pluzhnikov A Blumenthal MN Beaty TH Bleecker ER . Evidence for gene-environment interactions in a linkage study of asthma and smoking exposure. J Allergy Clin Immunol. (2003) 111:840–6. doi: 10.1067/mai.2003.170, PMID: 12704367

[11] Dizier MH Bouzigon E Guilloud-Bataille M Siroux V Lemainque A Boland A . Evidence for gene x smoking exposure interactions in a genome-wide linkage screen of asthma and bronchial hyper-responsiveness in EGEA families. Eur J Hum genetics: EJHG. (2007) 15:810–5. doi: 10.1038/sj.ejhg.5201830, PMID: 17426724

[12] Kuruvilla ME Lee FE Lee GB . Understanding asthma phenotypes, endotypes, and mechanisms of disease. Clin Rev Allergy Immunol. (2019) 56:219–33. doi: 10.1007/s12016-018-8712-1, PMID: 30206782 PMC6411459

[13] Kelchtermans J Mentch F Hakonarson H . Ambient air pollution sensitivity and severity of pediatric asthma. J Expo Sci Environ Epidemiol. (2023). doi: 10.1038/s41370-023-00573-7, PMID: 37369742 PMC10877545

[14] Kelchtermans J March ME Mentch F Qu H Liu Y Nguyen K . Genetic modifiers of asthma response to air pollution in children: An African ancestry GWAS and PM(2.5) polygenic risk score study. Environ Res. (2024) 267:120666. doi: 10.1016/j.envres.2024.120666, PMID: 39725137 PMC11800831

[15] Zhou W Yuan W Chen Y Li C Hu L Li Q . Single-cell transcriptomics reveals the pulmonary inflammation induced by inhalation of subway fine particles. J Hazardous Materials. (2024) 463:132896. doi: 10.1016/j.jhazmat.2023.132896, PMID: 37951166

[16] Líbalová H Uhlířová K Kléma J Machala M Šrám RJ Ciganek M . Global gene expression changes in human embryonic lung fibroblasts induced by organic extracts from respirable air particles. Particle Fibre Toxicol. (2012) 9:1. doi: 10.1186/1743-8977-9-1, PMID: 22239852 PMC3275518

[17] Lu X Li R Yan X . Airway hyperresponsiveness development and the toxicity of PM2.5. Environ Sci pollut Res. (2021) 28:6374–91. doi: 10.1007/s11356-020-12051-w, PMID: 33394441

[18] Ochoa D Hercules A Carmona M Suveges D Baker J Malangone C . The next-generation Open Targets Platform: reimagined, redesigned, rebuilt. Nucleic Acids Res. (2023) 51:D1353–d9. doi: 10.1093/nar/gkac1046, PMID: 36399499 PMC9825572

[19] McLaren W Gil L Hunt SE Riat HS Ritchie GRS Thormann A . The ensembl variant effect predictor. Genome Biol. (2016) 17. doi: 10.1186/s13059-016-0974-4, PMID: 27268795 PMC4893825

[20] Mountjoy E Schmidt EM Carmona M Schwartzentruber J Peat G Miranda A . An open approach to systematically prioritize causal variants and genes at all published human GWAS trait-associated loci. Nat Genet. (2021) 53:1527–33. doi: 10.1038/s41588-021-00945-5, PMID: 34711957 PMC7611956

[21] Ghoussaini M Mountjoy E Carmona M Peat G Schmidt EM Hercules A . Open Targets Genetics: systematic identification of trait-associated genes using large-scale genetics and functional genomics. Nucleic Acids Res. (2021) 49:D1311–D20. doi: 10.1093/nar/gkaa840, PMID: 33045747 PMC7778936

[22] Zhou Y Zhou B Pache L Chang M Khodabakhshi AH Tanaseichuk O . Metascape provides a biologist-oriented resource for the analysis of systems-level datasets. Nat Commun. (2019) 10:1523. doi: 10.1038/s41467-019-09234-6, PMID: 30944313 PMC6447622

[23] Kuleshov MV Jones MR Rouillard AD Fernandez NF Duan Q Wang Z . Enrichr: a comprehensive gene set enrichment analysis web server 2016 update. Nucleic Acids Res. (2016) 44:W90–7. doi: 10.1093/nar/gkw377, PMID: 27141961 PMC4987924

[24] Chen EY Tan CM Kou Y Duan Q Wang Z Meirelles GV . Enrichr: interactive and collaborative HTML5 gene list enrichment analysis tool. BMC Bioinf. (2013) 14:128. doi: 10.1186/1471-2105-14-128, PMID: 23586463 PMC3637064

[25] Xie Z Bailey A Kuleshov MV Clarke DJB Evangelista JE Jenkins SL . Gene set knowledge discovery with enrichr. Curr Protoc. (2021) 1:e90. doi: 10.1002/cpz1.90, PMID: 33780170 PMC8152575

[26] Zar JH . Biostatistical analysis, 4th ed. (1999).

[27] Hochberg Y Benjamini Y . More powerful procedures for multiple significance testing. Stat Med. (1990) 9:811–8. doi: 10.1002/sim.4780090710, PMID: 2218183

[28] Cohen J . A coefficient of agreement for nominal scales. Educ psychol measurement. (1960) 20:37–46. doi: 10.1177/001316446002000104

[29] Vazquez L Connolly. J . CHOP. Asthma. PheKB (2013). Available online at: https://phekb.org/phenotype/146. (Accessed December 1, 2025).

[30] Becht E McInnes L Healy J Dutertre C-A Kwok IW Ng LG . Dimensionality reduction for visualizing single-cell data using UMAP. Nat Biotechnol. (2019) 37:38–44. doi: 10.1038/nbt.4314, PMID: 30531897

[31] Aran D Looney AP Liu L Wu E Fong V Hsu A . Reference-based analysis of lung single-cell sequencing reveals a transitional profibrotic macrophage. Nat Immunol. (2019) 20:163–72. doi: 10.1038/s41590-018-0276-y, PMID: 30643263 PMC6340744

[32] Martens JH Stunnenberg HG . BLUEPRINT: mapping human blood cell epigenomes. Haematologica. (2013) 98:1487–9. doi: 10.3324/haematol.2013.094243, PMID: 24091925 PMC3789449

[33] Monaco G Lee B Xu W Mustafah S Hwang YY Carré C . RNA-seq signatures normalized by mRNA abundance allow absolute deconvolution of human immune cell types. Cell Rep. (2019) 26:1627–40.e7. doi: 10.1016/j.celrep.2019.01.041, PMID: 30726743 PMC6367568

[34] Chen Y Chen L Lun ATL Baldoni PL Smyth GK . edgeR v4: powerful differential analysis of sequencing data with expanded functionality and improved support for small counts and larger datasets. Nucleic Acids Res. (2025) 53. doi: 10.1093/nar/gkaf018, PMID: 39844453 PMC11754124

[35] Guo M Morley MP Jiang C Wu Y Li G Du Y . Guided construction of single cell reference for human and mouse lung. Nat Commun. (2023) 14:4566. doi: 10.1038/s41467-023-40173-5, PMID: 37516747 PMC10387117

[36] Gaddis N Fortriede J Guo M Bardes EE Kouril M Tabar S . LungMAP portal ecosystem: systems-level exploration of the lung. Am J Respir Cell Mol Biol. (2024) 70:129–39. doi: 10.1165/rcmb.2022-0165OC, PMID: 36413377 PMC10848697

[37] Keenan AB Torre D Lachmann A Leong AK Wojciechowicz ML Utti V . ChEA3: transcription factor enrichment analysis by orthogonal omics integration. Nucleic Acids Res. (2019) 47:W212–w24. doi: 10.1093/nar/gkz446, PMID: 31114921 PMC6602523

[38] Lachmann A Xu H Krishnan J Berger SI Mazloom AR Ma’ayan A . ChEA: transcription factor regulation inferred from integrating genome-wide ChIP-X experiments. Bioinf (Oxford England). (2010) 26:2438–44. doi: 10.1093/bioinformatics/btq466, PMID: 20709693 PMC2944209

[39] Chen W Pilling D Gomer RH . The mRNA-binding protein DDX3 mediates TGF-β1 upregulation of translation and promotes pulmonary fibrosis. JCI Insight. (2023) 8. doi: 10.1172/jci.insight.167566, PMID: 36821384 PMC10132153

[40] Cohen ML Brumwell AN Ho TC Garakani K Montas G Leong D . A fibroblast-dependent TGF-β1/sFRP2 noncanonical Wnt signaling axis promotes epithelial metaplasia in idiopathic pulmonary fibrosis. J Clin Invest. (2024) 134. doi: 10.1172/JCI174598, PMID: 38980870 PMC11405054

[41] Tang W Du L Sun W Yu Z He F Chen J . Maternal exposure to fine particulate air pollution induces epithelial-to-mesenchymal transition resulting in postnatal pulmonary dysfunction mediated by transforming growth factor-β/Smad3 signaling. Toxicol letters. (2017) 267:11–20. doi: 10.1016/j.toxlet.2016.12.016, PMID: 28041981

[42] Wu H Wang D Shi H Liu N Wang C Tian J . PM2.5 and water-soluble components induce airway fibrosis through TGF-β1/Smad3 signaling pathway in asthmatic rats. Mol Immunol. (2021) 137:1–10. doi: 10.1016/j.molimm.2021.06.005, PMID: 34175710

[43] Tang Y-N Ding W-Q Guo X-J Yuan X-W Wang D-M Song J-G . Epigenetic regulation of Smad2 and Smad3 by profilin-2 promotes lung cancer growth and metastasis. Nat Commun. (2015) 6:8230. doi: 10.1038/ncomms9230, PMID: 26354229

[44] Kraik K Tota M Laska J Łacwik J Paździerz Ł Sędek Ł . The role of transforming growth factor-β (TGF-β) in asthma and chronic obstructive pulmonary disease (COPD). Cells. (2024) 13:1271. doi: 10.3390/cells13151271, PMID: 39120302 PMC11311642

[45] Panek MG Karbownik MS Górski KM Koćwin M Kardas G Marynowski M . New insights into the regulation of TGF-β/Smad and MPK signaling pathway gene expressions by nasal allergen and methacholine challenge test in asthma. Clin Trans Allergy. (2022) 12. doi: 10.1002/clt2.12172, PMID: 35800124 PMC9250282

[46] Koćwin M Jonakowski M Przemęcka M Zioło J Panek M Kuna P . The role of the TGF-SMAD signalling pathway in the etiopathogenesis of severe asthma. Pneumonol Alergol Pol. (2016) 84:290–301. doi: 10.5603/PiAP.2016.0037, PMID: 27672072

[47] Tripathi P Deng F Scruggs AM Chen Y Huang SK . Variation in doses and duration of particulate matter exposure in bronchial epithelial cells results in upregulation of different genes associated with airway disorders. Toxicol Vitro. (2018) 51:95–105. doi: 10.1016/j.tiv.2018.05.004, PMID: 29753051 PMC6464127

[48] Deng Z Fan T Xiao C Tian H Zheng Y Li C . TGF-β signaling in health, disease and therapeutics. Signal Transduction Targeted Ther. (2024) 9. doi: 10.1038/s41392-024-01764-w, PMID: 38514615 PMC10958066

[49] Fichtner- Strober W Kawakami K Puri RK Kitani A . IL-13 signaling through the IL-13α2 receptor is involved in induction of TGF-β1 production and fibrosis. Nat Med. (2006) 12:99–106. doi: 10.1038/nm1332, PMID: 16327802

[50] Morath C Dechow C Lehrke I Haxsen V Waldherr R Floege J . Effects of retinoids on the TGF-beta system and extracellular matrix in experimental glomerulonephritis. J Am Soc Nephrol. (2001) 12:2300–9. doi: 10.1681/ASN.V12112300, PMID: 11675406

[51] Kim KI Kwon CI Lee JH Kim CD Yoon TJ . Inhibitory effect of mitoxantrone on collagen synthesis in dermal fibroblasts. Ann Dermatol. (2022) 34:206–11. doi: 10.5021/ad.2022.34.3.206, PMID: 35721328 PMC9171176

[52] Alman BL Pfister G Hao H Stowell J Hu X Liu Y . The association of wildfire smoke with respiratory and cardiovascular emergency department visits in Colorado in 2012: a case crossover study. Environ Health. (2016) 15. doi: 10.1186/s12940-016-0146-8, PMID: 27259511 PMC4893210

[53] Yamazaki S Shima M Yoda Y Oka K Kurosaka F Shimizu S . Exposure to air pollution and meteorological factors associated with children’s primary care visits at night due to asthma attack: case-crossover design for 3-year pooled patients. BMJ Open. (2015) 5:e005736. doi: 10.1136/bmjopen-2014-005736, PMID: 25941174 PMC4420953

[54] Villeneuve PJ Chen L Rowe BH Coates F . Outdoor air pollution and emergency department visits for asthma among children and adults: A case-crossover study in northern Alberta, Canada. Environ Health. (2007) 6:40. doi: 10.1186/1476-069X-6-40, PMID: 18157917 PMC2254596

[55] Ueda K Nitta H Odajima H . The effects of weather, air pollutants, and Asian dust on hospitalization for asthma in Fukuoka. Environ Health Prev Med. (2010) 15:350–7. doi: 10.1007/s12199-010-0150-5, PMID: 21432566 PMC2955906

[56] Sunyer J . Effect of nitrogen dioxide and ozone on the risk of dying in patients with severe asthma. Thorax. (2002) 57:687–93., PMID: 12149528 10.1136/thorax.57.8.687 PMC1746405

[57] Smargiassi A Kosatsky T Hicks J Plante C Armstrong B Villeneuve PJ . Risk of asthmatic episodes in children exposed to sulfur dioxide stack emissions from a refinery point source in Montreal, Canada. Environ Health Perspect. (2009) 117:653–9. doi: 10.1289/ehp.0800010, PMID: 19440507 PMC2679612

[58] Santus P Russo A Madonini E Allegra L Blasi F Centanni S . How air pollution influences clinical management of respiratory diseases. A case-crossover study in Milan. Respir Res. (2012) 13:95. doi: 10.1186/1465-9921-13-95, PMID: 23078274 PMC3511062

[59] Sacks JD Rappold AG Davis JA Jr. Richardson DB Waller AE Luben TJ . Influence of urbanicity and county characteristics on the association between ozone and asthma emergency department visits in North Carolina. Environ Health Perspect. (2014) 122:506–12. doi: 10.1289/ehp.1306940, PMID: 24569869 PMC4014762

[60] Orellano P Quaranta N Reynoso J Balbi B Vasquez J . Effect of outdoor air pollution on asthma exacerbations in children and adults: Systematic review and multilevel meta-analysis. PloS One. (2017) 12:e0174050. doi: 10.1371/journal.pone.0174050, PMID: 28319180 PMC5358780

[61] Pereira G Cook A De Vos AJ Holman CD . A case-crossover analysis of traffic-related air pollution and emergency department presentations for asthma in Perth, Western Australia. Med J Aust. (2010) 193:511–4. doi: 10.5694/j.1326-5377.2010.tb04034.x, PMID: 21034384

[62] Andersen ZJ Loft S Ketzel M Stage M Scheike T Hermansen MN . Ambient air pollution triggers wheezing symptoms in infants. Thorax. (2008) 63:710–6. doi: 10.1136/thx.2007.085480, PMID: 18267985

