# Supplementary material for: Genetic susceptibility to PM2.5 exposure and transcriptional responses in pediatric asthma: insights from single-cell transcriptomics
Source: Front Immunol. 2026 Feb 3;17:1644537. doi: 10.3389/fimmu.2026.1644537 (PMC12909167; doi:10.3389/fimmu.2026.1644537)
Supplement: Supplementary file 1 [file DataSheet1.docx]

# Data supplement: Genetic Susceptibility to PM_2.5_ Exposure Shapes Transcriptional Responses in Pediatric Asthma: Insights from Single-Cell Transcriptomics

# Supplementary methods description

## PM_2.5_ sensitivity polygenic risk score

As previously reported, our genotype data were generated on genotyping arrays from Illumina, using standard quality assurance cut-offs. ^1^ This included merging array versions within families on common single nucleotide polymorphisms (SNPs) and filtering for genotype missingness (geno 0.1), individual missingness (mind 0.02), and minor allele frequency (MAF >= 0.01), in that order using PLINK v1.9. ^2^ Furthermore, SNPs with MAF >= 0.05, R^2^ >= 0.3 were kept; for MAF < 0.05, R^2^ >= 0.5 were kept.

We used the Metascape and Enrichr tools to assess if any biological pathways are significantly enriched for members of this gene set. ^3-7^ Metascape’s default parameters were applied, retaining terms with a p-value < 0.01, a minimum count of 3, and an enrichment factor > 1.5. P-values were calculated using the cumulative hypergeometric distribution, and q-values were adjusted via the Benjamini-Hochberg procedure to account for multiple testing. ^8 9^

## Single cell RNA sequencing

Peripheral blood samples were collected from each participant using EDTA-coated tubes and were promptly processed at [*Institution censored for review*]. Peripheral blood mononuclear cells (PBMCs) were isolated using Ficoll density gradient centrifugation and subsequently stored in liquid nitrogen. Prior to sequencing, PBMCs were carefully thawed. The thawed cells were transferred into 50 mL of calcium- and magnesium-free 1X PBS and centrifuged at 500g for 5 minutes. The supernatant was discarded, and the cell pellet was resuspended in fresh 50 mL 1X PBS, followed by another centrifugation step at 500g for 5 minutes. Single-cell RNA sequencing was performed using the 10x Genomics Chromium GEM-X Single Cell 3' Reagent Kits v4, following the manufacturer's protocols. cDNA and library QC were performed using the Agilent Technologies 4200 TapeStation. Library QC was then confirmed by qPCR with the ABI Applied Biosystems ViiA 7. Sequencing was conducted on the Illumina NovaSeq 6000 using Flowcell S2 V1.5, 100 cycles, and 150 µL for GEX, utilizing SBS v4 chemistry. Data processing was carried out using the Cell Ranger 7.1.0 software suite (10x Genomics), with sequence reads aligned to the GRCh38 reference genome for transcript quantification and downstream analysis.

Data processing and analysis were performed using the Seurat R package, with SCTransform applied for normalization and scaling. ^10 11^ To enhance cross-sample comparability, datasets were aligned post-principal component analysis (PCA) using Harmony, enabling improved integration and batch effect correction. ^12^

# e-Figures


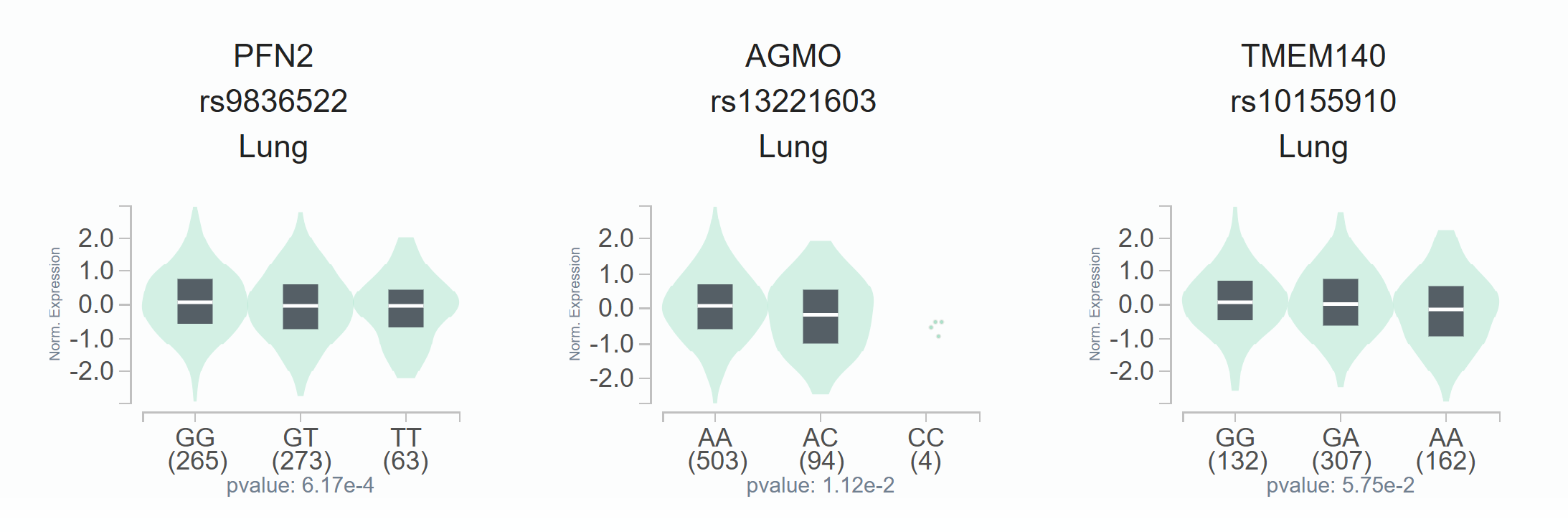


**e-Figure 1. Genotype-Stratified Expression of PFN2, AGMO, and TMEM140 in Lung Tissue.** We assessed whether any sPRS variants function as expression quantitative trait loci (eQTL) in lung tissue. The risk allele of rs9836522 was associated with higher PFN2 expression (metasoft meta-analysis P-value = 0.01), whereas the risk alleles for rs13221603 and rs10155910 were linked to lower AGMO and TMEM140 expression, respectively (metasoft meta-analysis P-value = 0.021 and 0.023 respectively). Violin plots display normalized gene expression across genotypes, illustrating the impact of these variants on transcript levels.

# e-Table legends:

**e-Table 1. Variant-to-Gene Mapping Results for sPRS Variants.** Variant-to-gene mapping was conducted using the V2G tool, which successfully linked 43 of the 44 variants in the sPRS to corresponding genes. The table details the variant identifiers, their assigned gene targets, and the Protein Atlas derived Protein Function.

**e-Table 2. Enrichment Analysis of GO:0043408 (Regulation of MAPK Cascade) for RUNX2 and PAX3-FKHR Target Genes.** Enrichment analysis was completed for target genes of both RUNX2 and PAX3-FKHR using Metascape. Metascape’s default parameters were applied, retaining terms with a p-value < 0.01, a minimum count of 3, and an enrichment factor > 1.5, with p-values computed via the cumulative hypergeometric distribution and q-values adjusted using the Benjamini-Hochberg procedure. Enriched terms were hierarchically clustered based on Kappa scores (similarity threshold > 0.3). This approach yielded significant enrichment for “GO:0043408 regulation of MAPK cascade”, with P = 4.56 x 10^-17^ for RUNX2 and P = 2.72 x 10^-11^ for PAX3-FKHR.

**e-Table 3. Cell-Level Differential Expression Across Immune Cell Subsets. .** Pair-wise cell-based differential expression analyses were performed as a secondary, exploratory analysis to provide biological context. Values for each matched case-control pair represent average log2 fold changes in gene expression.

**e-Table 4. Concordance Between Exploratory Cell-Level Gene Sets and Previously Reported PM2.5-Responsive Transcripts.** This table lists genes overlapping between exploratory cell-level gene sets from the present study and transcripts previously reported to respond to in vitro PM2.5 exposure. This analysis is intended to contextualize observed transcriptional patterns relative to prior PM2.5 studies and does not constitute formal validation of gene-level effects.

**e-Table 5. Exploratory Pathway and Process Enrichment Analysis.** This table summarizes pathway and biological process enrichment results derived from exploratory gene sets using Metascape. Analysis parameters included a p-value cutoff of 0.01, a minimum gene count of 3, and an enrichment factor >1.5, with multiple testing correction applied via the Benjamini-Hochberg procedure. Enriched terms were hierarchically clustered based on Kappa similarity scores (>0.3). These results are intended to highlight recurrent biological themes and support hypothesis generation rather than definitive pathway attribution.

# Supplementary references

1. Kelchtermans J, March ME, Mentch F, et al. Genetic modifiers of asthma response to air pollution in children: An African ancestry GWAS and PM(2.5) polygenic risk score study. *Environ Res* 2024;267:120666. doi: 10.1016/j.envres.2024.120666 [published Online First: 2024/12/27]

2. Chang CC, Chow CC, Tellier LC, et al. Second-generation PLINK: rising to the challenge of larger and richer datasets. *GigaScience* 2015;4:7. doi: 10.1186/s13742-015-0047-8 [published Online First: 2015/02/28]

3. Ghoussaini M, Mountjoy E, Carmona M, et al. Open Targets Genetics: systematic identification of trait-associated genes using large-scale genetics and functional genomics. *Nucleic acids research* 2021;49(D1):D1311-D20. doi: 10.1093/nar/gkaa840

4. Zhou Y, Zhou B, Pache L, et al. Metascape provides a biologist-oriented resource for the analysis of systems-level datasets. *Nat Commun* 2019;10(1):1523. doi: 10.1038/s41467-019-09234-6 [published Online First: 2019/04/05]

5. Kuleshov MV, Jones MR, Rouillard AD, et al. Enrichr: a comprehensive gene set enrichment analysis web server 2016 update. *Nucleic acids research* 2016;44(W1):W90-7. doi: 10.1093/nar/gkw377 [published Online First: 2016/05/05]

6. Chen EY, Tan CM, Kou Y, et al. Enrichr: interactive and collaborative HTML5 gene list enrichment analysis tool. *BMC Bioinformatics* 2013;14:128. doi: 10.1186/1471-2105-14-128 [published Online First: 2013/04/17]

7. Xie Z, Bailey A, Kuleshov MV, et al. Gene Set Knowledge Discovery with Enrichr. *Curr Protoc* 2021;1(3):e90. doi: 10.1002/cpz1.90 [published Online First: 2021/03/30]

8. Zar JH. Biostatistical Analysis. 4th ed1999.

9. Hochberg Y, Benjamini Y. More powerful procedures for multiple significance testing. *Statistics in medicine* 1990;9(7):811-8. doi: 10.1002/sim.4780090710 [published Online First: 1990/07/01]

10. Butler A, Hoffman P, Smibert P, et al. Integrating single-cell transcriptomic data across different conditions, technologies, and species. *Nature biotechnology* 2018;36(5):411-20.

11. Satija R, Farrell JA, Gennert D, et al. Spatial reconstruction of single-cell gene expression data. *Nature Biotechnology* 2015;33(5):495-502. doi: 10.1038/nbt.3192

12. Korsunsky I, Millard N, Fan J, et al. Fast, sensitive and accurate integration of single-cell data with Harmony. *Nature methods* 2019;16(12):1289-96.
